# FateNet: an integration of dynamical systems and deep learning for cell fate prediction

**DOI:** 10.1093/bioinformatics/btae525

**Published:** 2024-08-23

**Authors:** Mehrshad Sadria, Thomas M Bury

**Affiliations:** Department of Applied Mathematics, University of Waterloo, Waterloo, ON N2L 3G1, Canada; Department of Physiology, McGill University, Montreal, QC H3G 1Y6, Canada

## Abstract

**Motivation:**

Understanding cellular decision-making, particularly its timing and impact on the biological system such as tissue health and function, is a fundamental challenge in biology and medicine. Existing methods for inferring fate decisions and cellular state dynamics from single-cell RNA sequencing data lack precision regarding decision points and broader tissue implications. Addressing this gap, we present FateNet, a computational approach integrating dynamical systems theory and deep learning to probe the cell decision-making process using scRNA-seq data.

**Results:**

By leveraging information about normal forms and scaling behavior near bifurcations common to many dynamical systems, FateNet predicts cell decision occurrence with higher accuracy than conventional methods and offers qualitative insights into the new state of the biological system. Also, through in-silico perturbation experiments, FateNet identifies key genes and pathways governing the differentiation process in hematopoiesis. Validated using different scRNA-seq data, FateNet emerges as a user-friendly and valuable tool for predicting critical points in biological processes, providing insights into complex trajectories.

**Availability and implementation:**

github.com/ThomasMBury/fatenet.

## 1 Introduction

Complex dynamical systems can experience sudden shifts between states when they reach a critical threshold known as a tipping point or critical transition (Ambika and Kurths 2021). These transitions have been extensively studied in fields such as ecology, climate science, finance, and epidemiology (May *et al.* 2008, Jurczyk *et al.* 2017, Dakos *et al.* 2019, Drake *et al.* 2019, Dietz *et al.* 2021). Tipping points are also pertinent in the field of medicine, as seen in diseases such as diabetes (Li *et al.* 2014) and epileptic seizures (Maturana *et al.* 2020). These tipping points are characterized by a sudden shift from a healthy state to a diseased condition, signaling a critical change in the system’s dynamics (Chen *et al.* 2012). Recognizing early warning signs of these transitions during disease progression would enable the identification of pre-disease conditions and facilitate timely medical intervention (Meisel and Kuehn 2012). Fortunately, there are universal properties of tipping points that can present themselves before a tipping point occurs (Wissel 1984, Scheffer *et al.* 2009). One such example is critical slowing down, characterized by a decrease in local stability and systematic changes in properties of time series data such as variance, autocorrelation, and the power spectrum (Kleinen *et al.* 2003, Brock and Carpenter 2006). These universal properties suggest the possibility for early warning signals across a wide range of scientific domains (Dakos *et al.* 2008, Boettiger *et al.* 2013, Pace *et al.* 2017, Pananos *et al.* 2017, Boers 2018, Bury *et al.* 2020).

In developmental biology, cells undergo a variety of transitions as they differentiate. The Waddington landscape is a fundamental concept for understanding these critical transitions, as it explains how cells experience alterations in their transcriptome and epigenome while transitioning into unique cell types (Ferrell 2012). Predicting early cell fate bias and understanding the mechanisms underlying cell decision-making are crucial for advancing cellular reprogramming (Sadria and Layton 2023). By deciphering the regulatory mechanisms governing cell fate decisions, we can reprogram cells into specific cell types for regenerative medicine applications, such as replacing damaged or lost cells in various tissues and organs (Lin *et al.* 2018, Sadria *et al.* 2022a).

In recent years, single-cell technologies have provided us with a high level of precision in studying individual cells, allowing us to observe and understand cellular changes (Lee *et al.* 2020). However, they only provide a snapshot of the cellular state, limiting our ability to capture the dynamic changes that occur over time (Weinreb *et al.* 2018). To overcome this limitation, computational methods based on pseudotime analysis have been developed to reconstruct the temporal progression of cells (Ding *et al.* 2022). These methods infer the order of cells along a trajectory based on the similarity of their gene expression. Various techniques are employed to perform pseudotime analysis, such as using the distance to the root cell or computing entropy for each cell to position them in the trajectory (Saelens *et al.* 2019). While RNA velocity can be integrated into pseudotime analysis and provide information on the direction of cell state changes, it does not always provide accurate directions due to various factors (Gorin *et al.* 2022). Despite the promising results of all these methods, they are not yet able to detect critical transitions with high accuracy, and cannot provide specific details about the biological system changes that occur during and after the transition.

Cell fate transitions can be viewed as a bifurcation in a high-dimensional dynamical system (Ferrell 2012, Moris *et al.* 2016). It has been suggested that universal properties of bifurcations such as critical slowing down could be harnessed to provide early warning signals for their arrival (Scheffer *et al.* 2009). In a system subject to environmental and/or intrinsic noise, critical slowing down can be detected via an increase in variance and lag-1 autocorrelation, thereby serving as an early warning signal (Brock and Carpenter 2006, Dakos *et al.* 2008). Other early warning signals for bifurcations include an increase in entropy (Brett *et al.* 2017) and a decrease in Kolmogorov complexity (Dakos and Soler-Toscano 2016). These measures are currently used to predict bifurcations across a range of scientific domains (Dakos *et al.* 2023), including recent studies in cellular biology (Luo *et al.* 2022, Freedman *et al.* 2023, Zhong *et al.* 2023). However, they have had mixed success and are unable to predict the type of bifurcation.

In recent years, deep learning has emerged as a powerful tool for predicting changes in complex dynamical systems (Pathak *et al.* 2018, Deb *et al.* 2022, Bury *et al.* 2023, Dylewsky *et al.* 2023). In particular, a neural network can learn to predict bifurcations by training it on a massive corpus of simulation data from dynamical systems with noise (Bury *et al.* 2021). However, these current methods cannot be applied directly to pseudotime series of scRNA-seq data for two reasons: (i) scRNA-seq data is very high-dimensional, typically on the order of thousands of genes; and (ii) pseudotime series do not contain temporal correlations typical of dynamical systems with noise, since each data point is a snapshot from a unique cell, not a single cell evolving over time.

**Figure 1.**
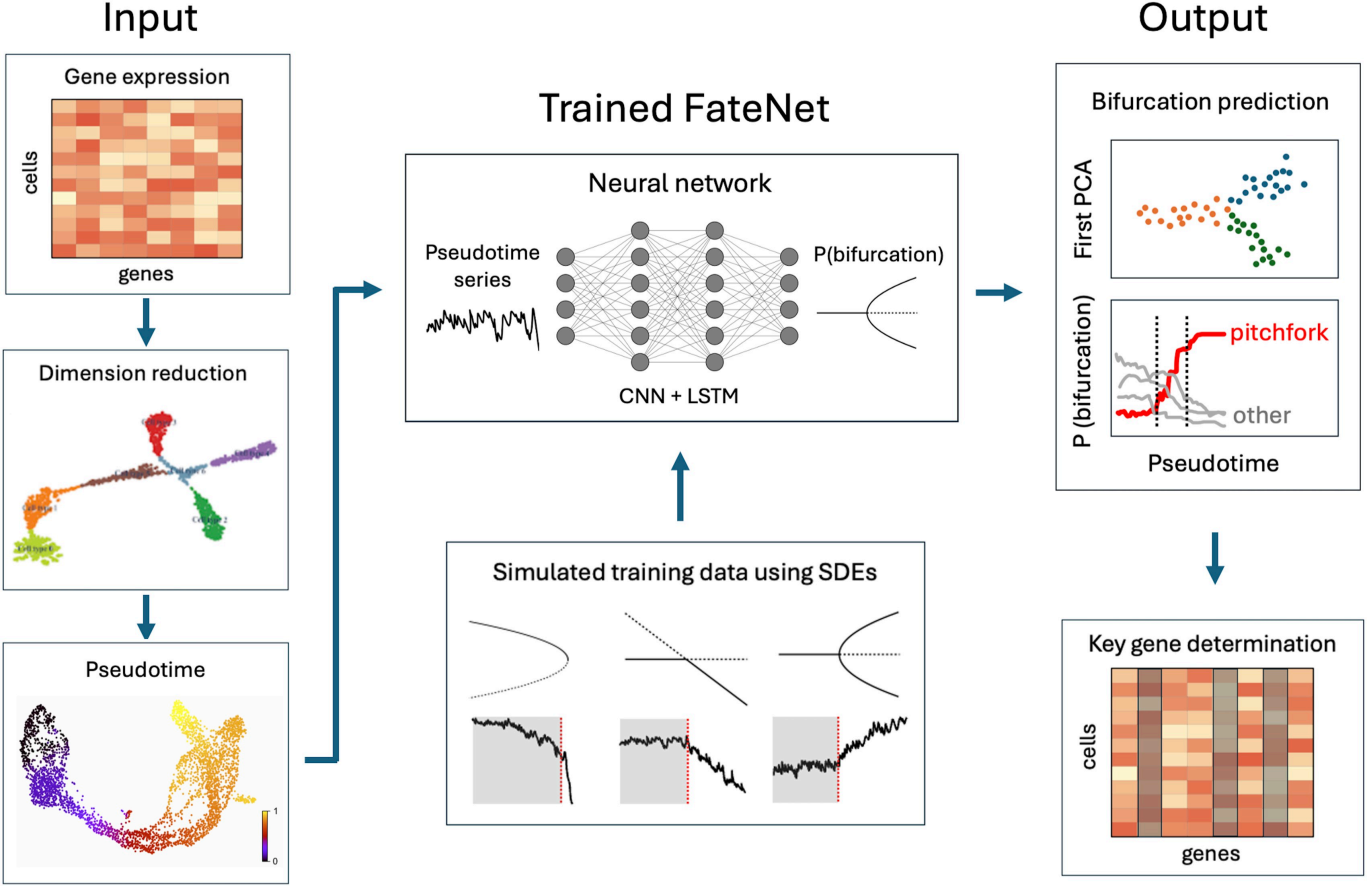
Schematic of workflow with FateNet. Input data, in the form of a gene expression matrix, is reduced in dimension using PCA, clusters are obtained and a pseudotime series is constructed. The pseudotime series leading up to a cell fate transition is passed into FateNet, which outputs a probability distribution over different bifurcations. FateNet is pre-trained using many simulations from stochastic differential equations (SDEs) going through different types of bifurcation. The output of FateNet consists of probabilities for the occurrence of different bifurcations. A spike in the probability for a bifurcation is an indication that the bifurcation is about to occur. The cells associated with the timing of the spike can be examined to identify the key genes responsible for initiating the bifurcation process

In this study, we introduce FateNet (Fig. 1), a novel computational model that combines the theory of dynamical systems and deep learning to predict cell fate decision-making using scRNA-seq data. By leveraging universal properties of bifurcations such as scaling behavior and normal forms (Kuznetsov 2004), FateNet learns to predict and distinguish different bifurcations in pseudotime simulations of a “universe” of different dynamical systems. The universality of these properties allows FateNet to generalize to high-dimensional gene regulatory network models and biological data. This approach not only provides an understanding of when cells undergo state changes but also captures the type of these transitions, identifying the characteristics of the system’s new state. By using FateNet we demonstrate how perturbing specific sets of genes can alter the type of transition a system undergoes. Notably, FateNet eliminates the need for training a model on the specific system under study and allows us to overcome the limitations of most deep learning models, which are typically restricted to the systems they were originally trained on. We test FateNet using simulated and biological scRNA-seq data of various sizes and compare its performance to current methods for bifurcation prediction. Our results demonstrate FateNet’s ability to detect the process of cell fate decision-making, offering insights into the ongoing transitions within the system and providing information on manipulating gene sets to modify transition types.

## 2 Materials and methods

### 2.1 Generation of training data for FateNet

We generate training data using simulations of a library of generated dynamical systems. Each dynamical system consists of the normal form for a bifurcation and higher-order polynomial terms with random coefficients and additive white noise. The higher-order terms add diversity to the training models, while still guaranteeing that they possess the desired bifurcation. We include fold, transcritical, and pitchfork bifurcations in the library.

The model framework for the fold bifurcation is

(1) $$x_{t+1}=-\mu +x_{t}-x_{t}^{2}+\sum_{i=3}^{10}\alpha_{i}{\left(x_{t}-\sqrt{-\mu }\right)}^{i}+\sigma \epsilon_{t},$$

where *x_t_* is the state variable, *μ_t_* is the (potentially time-dependent) bifurcation parameter, *α_i_* are drawn from the standard normal distribution, *σ* is the noise amplitude drawn from a uniform distribution between 0.005 and 0.015, and the noise process *ϵ_t_* is drawn from a standard normal distribution. The initial value for the bifurcation parameter *μ*_0_ is drawn from a uniform distribution with lower and upper bounds that make the dominant eigenvalue of Jacobian between 0 and 0.8 (the bifurcation occurs when this eigenvalue is 1). This results in bifurcation trajectories that start at different distances away from the bifurcation. Consequently, in the fold model, *μ*_0_ can take values between −0.25 and −0.01. In all models, the bifurcation occurs at *μ *= 0.

The model framework for the transcritical bifurcation is

(2) $$x_{t+1}=(1+\mu )x_{t}-x_{t}^{2}+\sum_{i=3}^{10}\alpha_{i}x_{t}^{i}+\sigma \epsilon_{t},$$

where *μ*_0_ can take values between −1 and −0.2. The model framework for the pitchfork bifurcation is

(3) $$x_{t+1}=(1+\mu )x_{t}\pm x_{t}^{3}+\sum_{i=4}^{10}\alpha_{i}x_{t}^{i}+\sigma \epsilon_{t},$$

where *μ*_0_ can take values between −1 and −0.2. For each model framework, we generate 1000 unique models. We run 20 simulations of each model going up to its bifurcation by incrementing *μ_t_* linearly from *μ*_0_ up to 0 over 600 time steps. If the model undergoes a noise-induced transition (defined as a deviation from equilibrium larger than 10 times the noise amplitude, *σ*), then the point prior to the transition is taken as the end of the time series. The last 500 data points are kept. If the model transitions before 500 points, it is discarded and replaced by a newly generated model. From these 20 simulations of 500 points, we construct 20 pseudotime series by placing the data in a 20 × 500 matrix and extracting the diagonal elements such that subsequent points in the pseudotime series come from different simulations. Formally, denoting the data point from simulation *i* at time *t* as $x_{i,t}$, for i=0,…,20,t=0,…,499, then the *i*th pseudotime series is given by $y_{i,t}=x_{(i+t)\;\mathrm{mod}\;20,t}$.

This process generates 20 000 “forced” pseudotime series for each bifurcation. We similarly generate 20 000 “null” pseudotime series where *μ* is kept fixed in each model. This gives a total of 80 000 time series that are labeled according to whether they are “fold,” “transcritical,” “pitchfork,” or “null” trajectories. This set of time series is then shuffled and partitioned into a training, validation and test set according to the ratio 0.95:0.025:0.025. The validation and test sets were chosen as a small percentage because a set containing a few thousand time series is adequate to provide a representative estimate of the performance measures used to assess the algorithm.

### 2.2 FateNet architecture and performance

FateNet consists of two types of neural network that are trained to predict a bifurcation label given a portion of pseudotime series data. Network 1 is trained on time series censored at the beginning and at the end by a randomly drawn length. This forces it to predict bifurcations based on the middle portions of the time series. Network 2 is trained on time series censored only at the beginning, allowing it to learn from data right up to the bifurcation. Formally, the length of the censored time series *L* is drawn from a uniform distribution with lower and upper bounds of 50 and 500, respectively. Then, for Network 1, the start time of the censored time series is drawn from a uniform distribution between 0 and 500-*L*, and for Network 2 the start time is set to 500-*L*. The censored time series are then normalized by their mean absolute value and prepended with zeros to make them 500 points in length—a requirement for the neural network. FateNet uses the average prediction from these two networks.

Each network has a CNN-LSTM (convolutional neural network-long short-term memory network) architecture (Supplementary Fig. S5). The CNN layers capture local features of the time series and LSTM layers capture dependencies over time. The networks receive an input time series $x\in R^{500}$ through a convolutional layer to obtain the hidden units

(4) $$h_{i}=\sigma \left(b+\sum_{j=1}^{12}w_{j}x_{i+j-6}\right)$$

where *i* runs from 0 to 500, *σ* is the ReLU activation function, and *b* and *w_j_* are the bias and weights of the kernel, which are trainable parameters. We use a kernel size of 12, and pad the edges of the time series with zeros to maintain the input dimension. We apply this operation for 50 kernel filters for Network 1 and 100 filters for Network 2. We then apply a dropout of 10%, which randomly fixes this proportion of hidden units to zero at each iteration of the training process. The units are then passed through a max pooling layer, which takes the maximum value over a window that strides over the units. We use a pool size of 2 and a stride length of 2. This process is then repeated for a second convolutional and max pooling layer.

The output is then passed to an LSTM layer with 100 memory cells for Network 1 and 50 cells for Network 2, where each cell is capable of capturing both long and short-term dependencies in the data (Hochreiter and Schmidhuber 1997, Gers *et al.* 2000). A LSTM cell consists of several components including the input gate $\left(i_{t}\right)$, the forget gate $\left(f_{t}\right)$, the output gate $\left(o_{t}\right)$, the cell state $\left(c_{t}\right)$ and the hidden state $\left(h_{t}\right)$, where *t* runs from 0 to 250. Each LSTM cell is updated as follows. The forget, input, and output gates are update as

$$\begin{array}{c} f_{t}=\sigma \left(W_{f}x_{t}+U_{f}h_{t-1}+b_{f}\right)\\ i_{t}=\sigma \left(W_{i}x_{t}+U_{i}h_{t-1}+b_{i}\right)\\ o_{t}=\sigma \left(W_{o}x_{t}+U_{o}h_{t-1}+b_{o}\right) \end{array}$$

where *x_t_* is the input from the previous hidden layer, *W* and *U* are weight matrices, *b* are bias vectors, *σ* is the sigmoid activation function

$$\sigma (x)=\frac{e^{x}}{1+e^{x}},$$

and the initial value for the hidden state is $h_{0}=0$. Meanwhile, a cell input activation vector is computed as

$${\tilde{c}}_{t}=\tanh\left(W_{c}x_{t}+U_{c}h_{t-1}+b_{c}\right)$$

which is used to determine the new cell state and hidden state

(5) $$c_{t}=f_{t}*c_{t-1}+i_{t}*{\tilde{c}}_{t}$$

(6) $$h_{t}=o_{t}*\tanh\left(c_{t}\right)$$

where * is element-wise multiplication of vectors. The initial value for the cell state is taken as $c_{0}=0$.

This is then passed to the second LSTM layer with 20 memory cells. Here, only the final value of each cell state sequence is stored. Finally, this is passed through a dense layer with softmax activation to obtain the probabilities

$$y_{i}=\mathrm{softmax}\left(b_{i}+\sum_{j=1}^{10}w_{ij}x_{j}\right)$$

where *i* runs from 1 to 4, *b_i_* are the biases, $w_{ij}$ are the weights, and *x_j_* are the inputs from the previous hidden layer. In total, Network 1 contains 100 864 trainable parameters and Network 2 contains 157 364. To obtain optimal values, we performed a hyperparameter sweep over the number of convolutional layers, the number of convolutional filters, and the number of memory cells in the LSTM layers.

The networks were initialized and trained in Tensorflow v 2.10 using the Adam optimization algorithm with a learning rate of 0.0005, a batch size of 1024, and categorical cross entropy as a loss function, given by

$$L=-\sum_{i=1}^{4}t_{i}\;\text{log}\;\left(y_{i}\right)$$

where the vector *t* is the one-hot encoded truth label and *y* is the probability vector output from the model. The networks reached peak performance on their validation sets after 71 and 154 epochs, respectively (Supplementary Fig. S6). When evaluated on the test set, Network 1 obtained an F1 score of 0.64 on the multi-class prediction problem and 0.80 on the binary prediction problem. Network 2 obtained F1 scores of 0.95 and 0.98, respectively. The lower performance of Network 1 is due to it being trained and tested only on middle portions of time series that do not necessarily contain distinguishable information about the type of bifurcation. The confusion matrices (Supplementary Fig. S7) show the performance of the networks at predicting each class in the test set. Out-of-sample predictions are made by taking the average prediction across an ensemble of 5 networks of type 1 and 5 networks of type 2.

### 2.3 Applying the model to pseudotime data

To obtain model predictions on pseudotime data, we first obtain the principal component from a PCA. We then detrend it using a Lowess filter with a span of 0.2. Predictions from our model at a given point in pseudotime are obtained by taking the preceding data, normalizing them, prepending them with zeros to make the input 500 points in length, and feeding it into the model. The model then outputs a probability vector, whose components indicate the probability of each event. These computations are performed with the Python package ewstools (Bury 2023).

### 2.4 Alternative methods for bifurcation prediction

We compare the performance of FateNet with popular methods such as variance, lag-1 autocorrelation, sample entropy and Kolmogorov complexity. An increase in the first three and a decrease in the latter is expected prior to the bifurcation. We compute these measures on the detrended data over a rolling window of 0.25 according to standard methods (Dakos *et al.* 2012). The sample entropy and Kolmogorov complexity are computed using EntropyHub (Flood and Grimm 2021). The Kendall tau value is used to assess the trend of the measures, and as a discrimination threshold for computing the ROC curves.

### 2.5 Simple model for a gene regulatory network

This model consists of two coupled differential equations (Freedman *et al.* 2023) adapted to include additive Gaussian white noise and an additional parameter to modulate the nonlinear interaction between the genes. The model is given by

$$\begin{array}{cc} & {\dot{g}}_{1}=\frac{m_{1}}{\left(1+g_{2}^{2}\right)}-k_{D}g_{1}+\sigma \xi_{1}(t)\\ & {\dot{g}}_{2}=\frac{m_{2}}{\left(1+cg_{1}^{2}\right)}-k_{D}g_{2}+\sigma \xi_{2}(t) \end{array}$$

where *g*_1_ and *g*_2_ are the level of gene expression for each gene, *k_D_* is the degradation rate, *m*_1_ and *m*_2_ determine the scales of their synthesis, c governs the nonlinear response of gene 2 to gene 1,σ is the noise amplitude, and $\xi_{1}(t)$ and $\xi_{2}(t)$ are Gaussian white noise processes.

The model possesses a fold and a pitchfork bifurcation. We simulate trajectories going through both types of bifurcation, as well as a null trajectory where there is no bifurcation but still a time-dependent parameter. For the fold trajectory, we take *m*_1_ increasing linearly from 1 to $4.75,m_{2}=3,k_{D}=1$, and *c *=* *1. For the pitchfork trajectory, we take $m_{1}=1,m_{2}=1,k_{D}$ decreasing linearly from 1 to 0.25, and *c *=* *1. For the null trajectory, we take the same parameters as the fold trajectory, except we set *c *=* *0.1, which removes the fold bifurcation. In each case, we simulate the model using the Euler-Maruyama method with a step size of 0.01 and a noise amplitude σ=0.05. We then down-sample the data by a factor of 100. As described in the section on generating training data, we then construct a pseudotime series using 20 simulations of the model and taking subsequent points from each simulation.

### 2.6 SERGIO simulation

SERGIO is a simulator of single-cell gene expression data based on a stochastic differential equation (SDE) framework that can simulate noise and variability of gene expression, as well as the effects of external stimuli and cell cycle progression. SERGIO can model the stochastic nature of transcription, the regulation of genes by multiple transcription factors, and the differentiation of cells along complex trajectories. SERGIO also allows users to specify a gene regulatory network and various parameters that control the simulation process. Using SERGIO, we generated a synthetic scRNA-seq dataset that mimics a dynamic differentiation program, similar to the DS11 dataset described by (Dibaeinia and Sinha 2020). Our dataset consists of 1800 single cells from 7 cell types, with a gene regulatory network of 100 genes.

### 2.7 Analysis of scRNA-seq data

As a preprocessing step, we scaled, centered, and log-normalized the scRNA-seq gene expression data, and extracted the top 3000 most variable genes. We used the top 40 principal components to map the highly variable genes onto a lower dimension for clustering, using a k-nearest-neighbor graph with *K* = 30. Clusters were visualized using the Python package “UMAP” (McInnes et al., 2018). For the scRNA-seq data, partition-based graph abstraction (PAGA) was employed to sort cells accurately over time, allowing for a detailed mapping of the dynamic progression of the processes (Wolf *et al.* 2019). PAGA is an extended version of diffusion pseudotime that considers disconnected graphs. By covering both aspects of clustering and pseudotemporal ordering it can model the underlying biological progression, assigning each cell a pseudotime value that reflects its position along the inferred trajectory. Also, Cytoscape and Enrichment Map are used to investigate and visualize the connection between the identified biological pathways (Merico *et al.* 2010, Franz *et al.* 2023).

In testing our model, we consider the bifurcation from undifferentiated cells to neutrophils. The bifurcation is crossed at approximately pseudotime 0.6. For making predictions, we consider the first PCA component as a function of pseudotime up to the bifurcation. This still contains a lot of data (61 309 cells), so we down-sample by a factor of 100 to obtain a shorter time series more appropriate for our model. We then detrend the data using a Lowess filter with a span of 0.2. We are able to obtain 100 unique bifurcation trajectories from the biological data by shifting the down-sampling procedure one point at a time. We construct 100 null time series (that do not undergo a bifurcation) by sampling randomly from the first 20% of the detrended data and adding it to the trend of the original data.

To investigate the effect of gene knockout, we set the expression of a fixed number of genes to zero. The genes that are selected are those that are most highly represented in the top PCA component. Once these gene expressions have been set to zero, we recompute the top PCA component to obtain a new bifurcation trajectory. To investigate the effect of gene overexpression, we follow a similar procedure, except we multiply the expression of the most significant genes by a factor of two.

## 3 Results

### 3.1 Bifurcation prediction in a simple gene regulatory network

**Figure 2.**
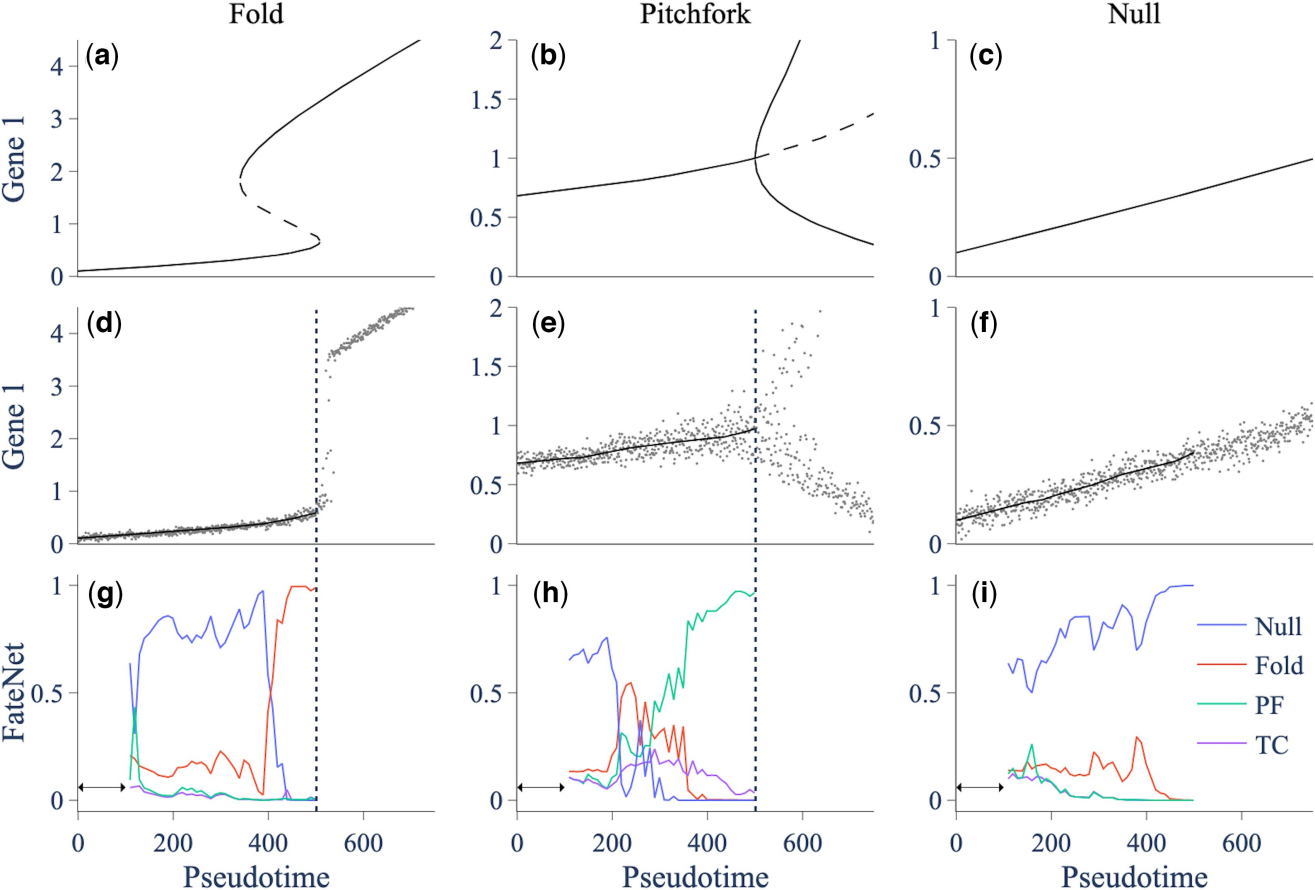
Simulations and predictions in the simple gene regulatory network model going through a fold, pitchfork, and no bifurcation. (a–c) Bifurcation diagrams showing the stable (solid) and unstable (dashed) states of the model as a parameter is varied. (d–f) Model simulation (gray) with the bifurcation parameter varying linearly with time (Section 2), and smoothing (black) with a Lowess filter with span 0.2. The model reaches the bifurcation at pseudotime 500. (g–i) Probabilities assigned by FateNet for each class of bifurcation as progressively more of the data becomes available. The arrow shows the time window where there is insufficient data for FateNet to make a prediction. FateNet uses the data after smoothing (i.e. not the trend) when making its predictions. The vertical dashed line indicates the time when the bifurcation is crossed. PF: pitchfork; TC: transcritical.

To demonstrate FateNet, we use data generated from a simple model of a gene regulatory network that can undergo different types of bifurcation as different parameters are varied (Section 2). The model undergoes a fold bifurcation as the synthesis rate of the first gene increases (*m*_1_), and a pitchfork bifurcation as the degradation rate of the genes (*k_D_*) decreases (Freedman *et al.* 2023). We simulate the model with additive white noise and a linearly changing parameter that leads to (i) a fold bifurcation, (ii) a pitchfork bifurcation, and (iii) no bifurcation (Fig. 2). In the bifurcation scenarios, the bifurcation is reached at pseudotime 500. At a given point in time, FateNet takes in all preceding data and assigns a probability for a fold, transcritical and pitchfork bifurcation, and a probability for no bifurcation (null). A heightening in a bifurcation probability is taken as a signal that this bifurcation is approaching. FateNet successfully signals the approach of a fold and a pitchfork bifurcation in the gene regulatory network. In the case of the null trajectory, no significant probability is assigned to any of the bifurcations. It also signals the correct bifurcations in the cases of larger and smaller noise (Supplementary Figs S1 and S2).

### 3.2 Bifurcation prediction in a large simulated gene regulatory network

**Figure 3.**
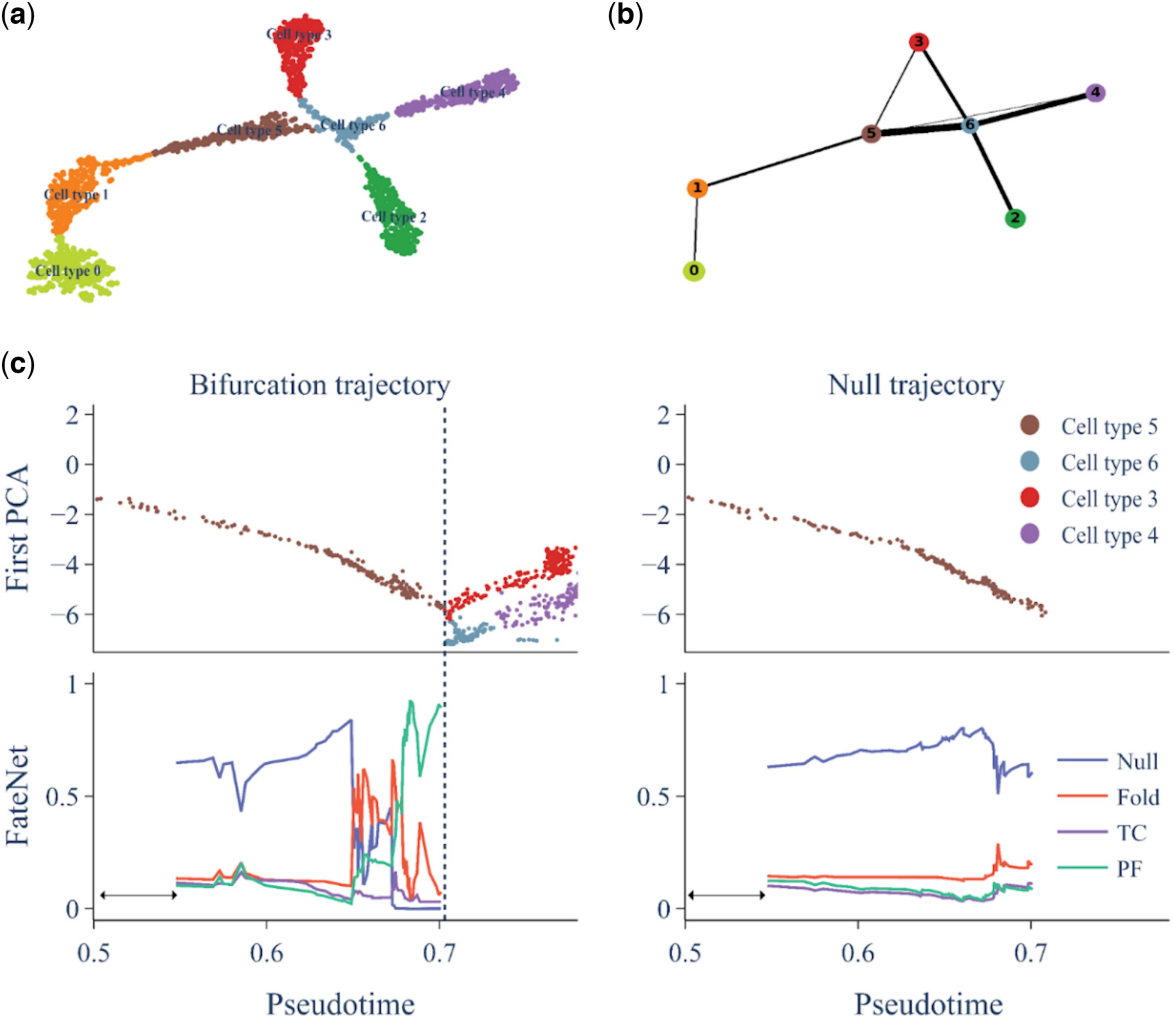
Bifurcation predictions in a simulation of SERGIO. (a) Uniform Manifold Approximation and Projection (UMAP) visualization of scRNA-seq data generated by SERGIO, with distinct clusters, color-coded based on cell type. (b) PAGA network graph representing the interconnectivity and relationships between cell types. (c) Bifurcation and null trajectories of cells organized in pseudotime (top) and the predictions of FateNet (bottom). The first principal component of the gene expression data is used to make predictions. The bifurcation trajectory shows a cell-fate transition between cell type 5 and cell types 3 and 6. The vertical dashed line indicates the time when the bifurcation is crossed. Data is smoothed using a Lowess filter with span 0.2 and the detrended data are passed to our model. The null trajectory is generated by taking a random sampling from the first 20% of the detrended data and adding it to the original trend. DL probabilities are the probabilities assigned by our model for each event among Null, Fold, Transcritical (TC), and Pitchfork (PF).

To assess the performance of FateNet on larger datasets we use SERGIO, a simulator based on stochastic differential equations that can generate both steady-state and dynamic scRNA-seq data (Dibaeinia and Sinha 2020). It incorporates different noise types for realistic dataset creation. We generate differentiation simulation data with 1800 cells, 100 genes, and 7 cell types (Fig. 3a). After the preprocessing steps in the 100D gene space, we apply Principal Component Analysis (PCA) to reduce the dimensionality of the data and obtain the principal components. To establish a cell ordering and understand the pseudotime dynamics within this dataset, we use Partition-based graph abstraction (PAGA) (Fig. 3b) (Wolf *et al.* 2019). These data are then detrended and used as input to FateNet, leveraging the known underlying differentiation trajectory.

Focusing on one of the bifurcation points, our objective is to test whether our model can predict this bifurcation in advance and identify the specific type of bifurcation occurring. We find that FateNet not only provides an early signal for the upcoming change in cell state but also successfully identifies a pitchfork bifurcation in advance, consistent with the observed change in state at the cell-fate transition (Fig. 3c, bifurcation trajectory panel). We then test our model on a scenario where the system does not undergo a cell-fate transition (Fig. 3c, null trajectory panel). To generate such a trajectory, we sample points randomly (with replacement) from the first 20% of the detrended data and add it to the trend of the original data. In this case, our model correctly predicts the absence of any critical transition (Null), indicating that it has learned distinct features associated with the presence/absence of an upcoming cell-fate transition (Fig. 3c, null trajectory panel).

### 3.3 Bifurcation prediction in biological data

**Figure 4.**
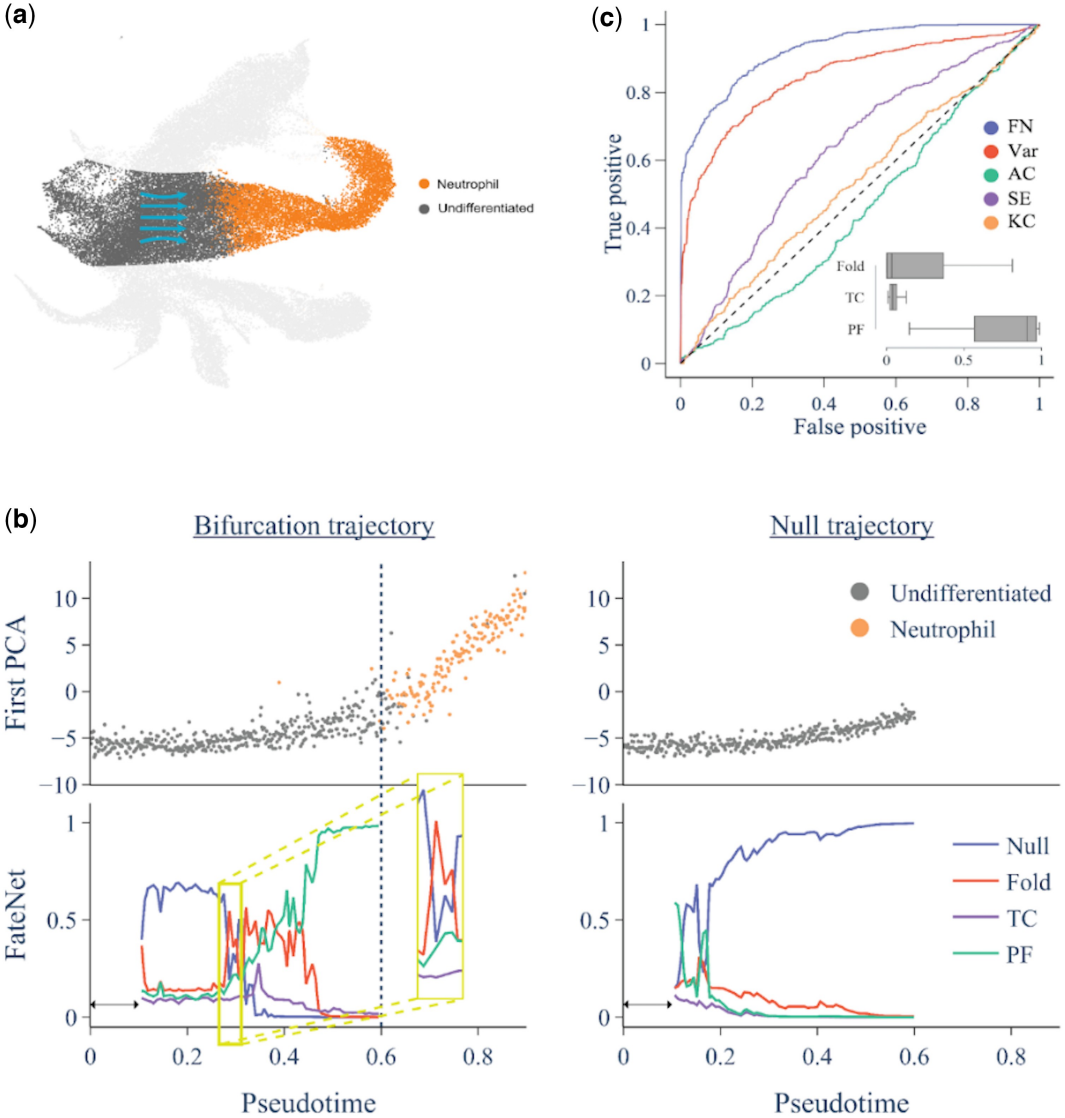
Predictions in data of mouse hematopoietic stem cell differentiation from undifferentiated cells (gray, left part) to neutrophils (orange, right part). (a) UMAP plot of mouse hematopoiesis data, emphasizing the transition (arrows) from progenitor cells (gray, left part) to neutrophils (orange, right part), elucidating the dynamic differentiation process. (b) Bifurcation and null trajectories with model predictions. The bifurcation trajectory (left) is the first principal component against pseudotime down-sampled by a factor of 100. The dashed line shows the transition. The data is detrended using a Lowess filter with a span 0.2 and used as input to the model. The model outputs probabilities for each event among Null, Fold, Transcritical (TC), and Pitchfork (PF). The yellow box highlights the initial spike in bifurcation probabilities between pseudotime 0.28 and 0.32. The null trajectory (right) is generated by random sampling from the first 20% of the detrended bifurcation trajectory and adding them to the trend. (c) ROC curves for predictions of any bifurcation using variance (Var), lag-1 autocorrelation (AC), sample entropy (SE), Kolmogorov complexity (KC) and FateNet (FN). Predictions are made at evenly spaced time points between 0.3 and 0.6 for 100 unique down-sampled bifurcation trajectories and corresponding nulls, resulting in a total of 1400 predictions. The inset shows the probabilities assigned to each bifurcation between pseudotime 0.5 and 0.6. Boxes show the median and interquartile range, and whiskers show the full range.

To test FateNet on biological data, we use temporal scRNA-seq data of mouse hematopoietic stem cell differentiation with 130 887 cells and 25 289 genes (Weinreb *et al.* 2020). Our emphasis is on the differentiation of progenitor cells, specifically exploring the decision-making process of neutrophil fate (Fig. 4a). In this context, we aim to understand both the timing of cell fate decision-making and the specific type of differentiation occurring within the system. Therefore, we use cells that are classified as undifferentiated or neutrophils and extract the top 3000 most variable genes. On this, PCA is conducted and the first principal component is used to make predictions of a bifurcation (Fig. 4b, bifurcation trajectory panel). We find that FateNet predicts a pitchfork bifurcation before the transition from an undifferentiated cell to a neutrophil. The transition is also preceded by an increase in variance, which is consistent with the phenomenon of critical slowing down that accompanies bifurcations. We compare this with a null time series that is generated by taking a random sample from the first 20% of the detrended bifurcation trajectory and adding this to the trend. This way, we demonstrate that the model is not making predictions based on the trend, but rather on the dynamics around the trend, which provide information about an approaching bifurcation. On the null trajectories, our model correctly predicts “Null,” i.e. no bifurcation (Fig. 4b, null trajectory panel).

To assess the performance of FateNet and compare it to conventional methods for bifurcation prediction, we make predictions on 100 unique down-sampled bifurcation trajectories and corresponding nulls from the biological data. For each trajectory, we make seven equally spaced predictions between pseudotime 0.3 and 0.6, resulting in a total of 1400 predictions. The receiver operating characteristic (ROC) curve (Fig. 4c) illustrates performance on the binary classification problem of whether or not a bifurcation is approaching. An area under the curve (AUC) of 1 corresponds to a perfect performance, whereas an AUC of 0.5 (dashed line) is no better than random. FateNet achieves the highest performance (AUC = 0.93), followed by variance (AUC = 0.85), sample entropy (AUC = 0.63), Kolmogorov complexity (AUC = 0.53), and lag-1 autocorrelation (AUC = 0.46). In addition, FateNet uniquely provides a prediction about the bifurcation type, which becomes more evident closer to the bifurcation. We show the specific bifurcation probabilities from pseudotime 0.5 onwards, demonstrating that pitchfork is the favored bifurcation across the 100 down-sampled trajectories.

To understand the underlying biological mechanisms governing cell fate decision-making, we focus on a critical segment of the pseudotime trajectory between 0.28 and 0.32, where a notable increase in bifurcation probability occurs (Fig. 4b, yellow box). By conducting differential gene expression analysis on cells within this specific temporal window, we identify key genes such as Myc, Ybx1, S100a8, and S100a9, Set, and H2afy whose expression showed a significant change compared to other parts of the trajectory. Remarkably, several of these genes have been shown by previous studies to play a key role in fundamental cellular processes, including stem cell differentiation, regulation of neutrophil differentiation, chromatin remodeling, and cellular metabolism (Supplementary Table S1 for a detailed list of genes, their functions and reference to previous studies). Furthermore, we leverage the top 250 genes with significant expression changes, from our results to scrutinize cellular pathways, components and functions involved in cell decision-making. Our analysis reveals enrichment in pathways linked to metabolic processes (organonitrogen compound metabolism, catabolic process, superoxide anion generation, protein metabolic processes), cell death, protein localization, and leukocyte activation (Supplementary Fig. S3). These findings align with existing literature showing hematopoietic stem cells navigate a complex array of developmental pathways, including not only self-renewal and differentiation but also apoptosis and metabolism. The ultimate fate of dividing stem cells is shaped by the combination of signals from various regulators. Additionally, we use the enrichment map analysis to show the network of enriched pathways, illustrating the complex relationships and communication between the identified biological processes (Supplementary Fig. S4). This result not only can help us understand the connections between active pathways within the cell’s environment but also emphasizes the dynamic interactions influencing different regulatory mechanisms of cell fate decision making.

**Figure 5.**
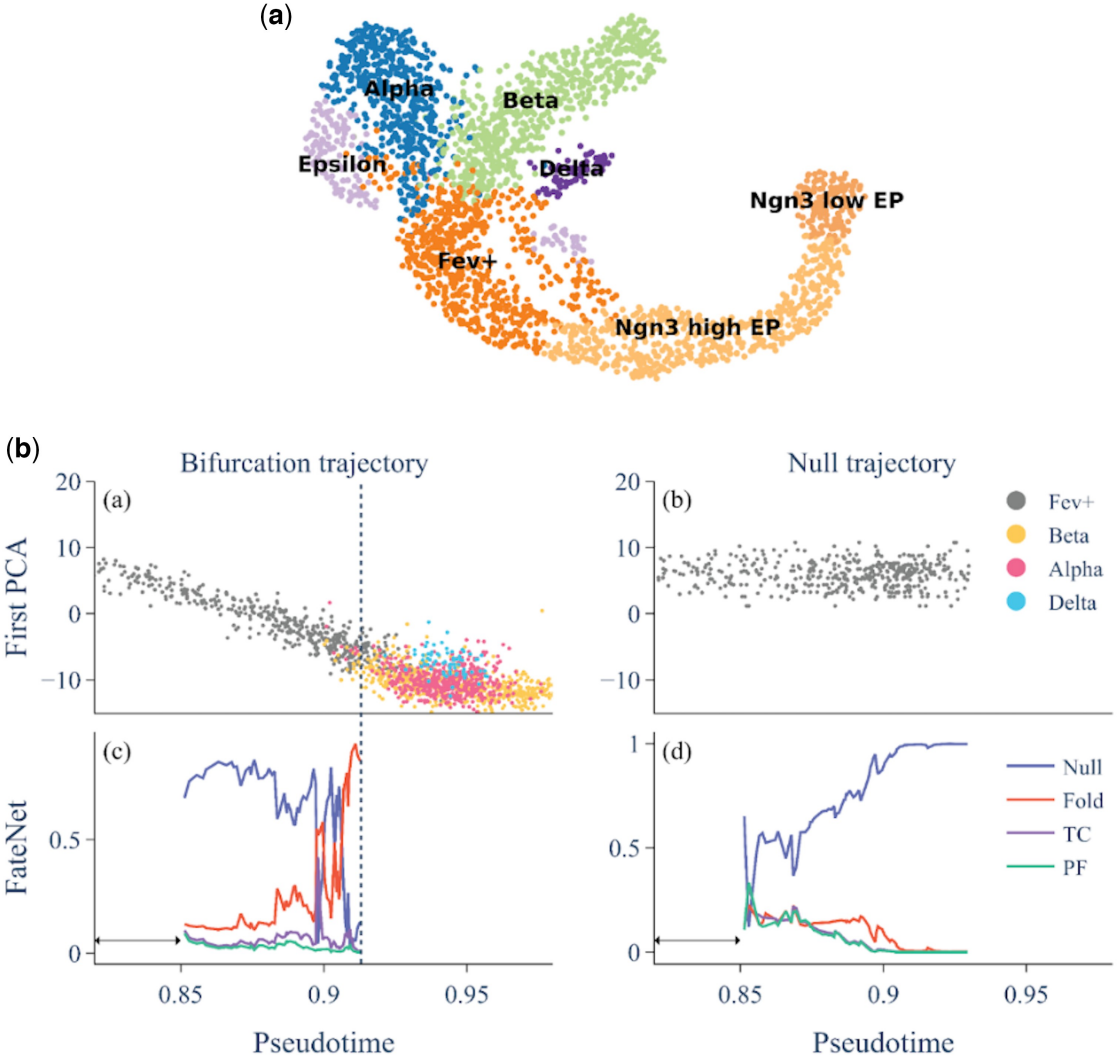
Bifurcation predictions in murine pancreatic development focusing on differentiation of Fev+ to Alpha, Beta and Delta cells. (a) Uniform Manifold Approximation and Projection (UMAP) visualization of Pancreas development data, with distinct clusters, color-coded based on cell type. (b) Bifurcation and null trajectories with model predictions. The bifurcation trajectory (left) is the first principal component against pseudotime. The dashed line shows the transition point. The data is detrended using a Lowess filter with a span of 0.2 and used as input to the model. The model outputs probabilities for each event among Null, Fold, Transcritical (TC), and Pitchfork (PF). The null trajectory (right) is generated by random sampling from the first 20% of the detrended bifurcation trajectory and adding them to the trend.

To further validate our findings, we conduct an analysis on a mouse pancreas scRNA-seq dataset obtained from embryonic day 15.5 (Bastidas-Ponce *et al.* 2019), comprising 2531 cells categorized into seven distinct cell types (Fig. 5a). Our primary objective is to focus on the differentiation process among endocrine cells. Through pseudotime ordering, we delineate a trajectory originating from Fev+ cells, which subsequently bifurcated into multiple main branches. The termini of these branches include differentiated Alpha, Beta and Delta cells, indicating that the branches represent a transition toward fully differentiated cell states.

FateNet predicts a fold bifurcation for the transition from Fev+ cells to Alpha, Beta and Delta cells, identifying this bifurcation in advance at around 0.89 (Fig. 5b, bifurcation trajectory panel). To further test FateNet’s accuracy, we compared it with a null time series generated by taking a random sample from the first 20% of the detrended bifurcation trajectory and adding this to the trend. FateNet correctly predicts “Null,” indicating no bifurcation in this control scenario (Fig. 5b, null trajectory panel).

### 3.4 Effect of gene knockout/over-expression

**Figure 6.**
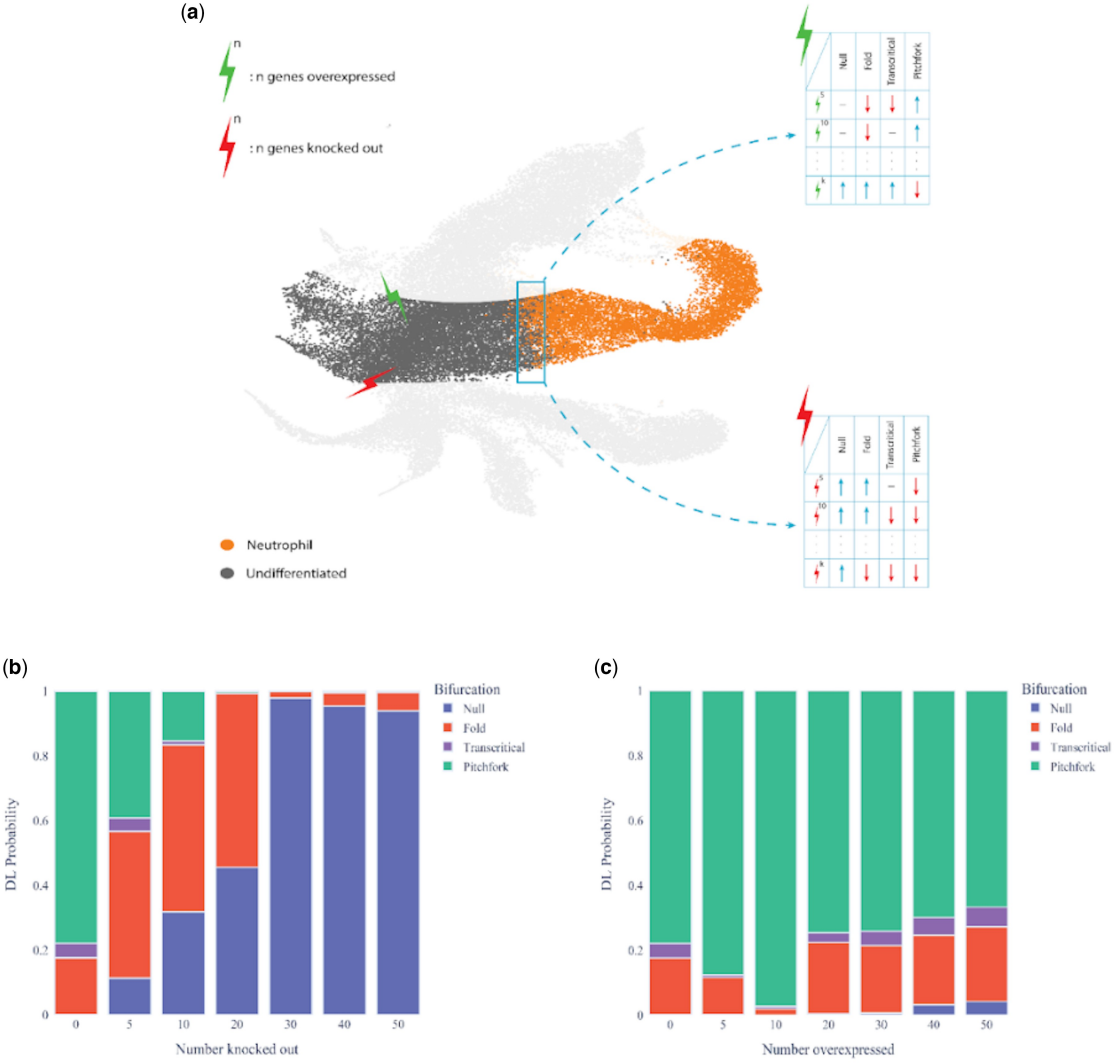
Exploring system response to various *in silico* perturbations. (a) UMAP visualization shows perturbations, with green lightning (upper) indicating overexpression and red lightning (lower) denoting knockout perturbations. Each perturbation is individually implemented to observe how the system experiences shifts in the bifurcation dynamics. Model predictions for stem cell differentiation to neutrophils in mouse hematopoiesis after knocking out (b) and over-expressing (c) a few numbers of the most significant genes. Genes are knocked out by setting their expression to zero. Genes are overexpressed by multiplying their expression by a factor of two. In each case, ten equally spaced predictions are made between pseudotime 0.45 and 0.6.

We investigate the effect of *in silico* knocking out and overexpressing genes (hard and soft interventions) on the predictions made by our model (Fig. 6a). We knockout the most significant genes in the first PCA component of the data by setting their expression to zero. We find that knocking out as few as five of the top genes results in a change in the bifurcation prediction from a pitchfork bifurcation to a fold bifurcation (Fig. 6b). Continuing to knock out genes increases the prediction for no bifurcation (Null) until eventually, after knocking out the top 30 genes, no bifurcation is predicted at all. We also overexpress genes by multiplying their expression by a factor of two. We find that overexpressing a small number of the top genes (5–10) strengthens the prediction of a pitchfork bifurcation (Fig.6c), whereas overexpressing a larger number of genes weakens the prediction of a pitchfork bifurcation. These results suggest that there are a few genes that are instrumental in the type of bifurcation that the system goes through. When these key genes are subjected to knockout, there is a substantial alteration in the bifurcation type of the system’s dynamics (Fig. 6b). On the other hand, with the top genes overexpressed, the bifurcation type is predicted with greater probability (Fig. 6c). However, over-expressing a broader set of genes can trigger additional regulatory mechanisms, resulting in an increased probability for other types of bifurcations.

## 4 Discussion

In various domains such as ecology, climate, health, and finance, the identification of critical points, often referred to as bifurcation points, holds significant importance. Early detection is crucial for strategic decision-making and intervention, minimizing the potential for adverse consequences. Also understanding the type of these transitions is pivotal for preemptive actions in these diverse fields (Clements *et al.* 2019). For instance, in health, the heart can spontaneously transition from a normal rhythm to a dangerous one, known as a cardiac arrhythmia. Early detection of these critical transitions in cardiac activity can enable prompt medical intervention, significantly impacting patient outcomes and preventing life-threatening situations (Glass and Mackey 2020).

Biological processes exhibit similar critical points and can undergo different types of transitions which occur during both normal development and disease progression. In normal developmental trajectories, cells move through Waddington’s landscape, experiencing bifurcations regulated by genetic and environmental cues. These events shape cell fate, determining whether a cell adopts a neuronal, muscular, or another specialized identity (Zhou and Huang 2011). Similarly, in disease trajectories, bifurcations can lead to different outcomes. For instance, consider a disease process where a cell can either recover or progress to a more severe state (Goldbeter 2018). Analyzing the paths cells take during differentiation and the process of cellular decision-making, including the precise timing of these events, is crucial for understanding development and unlocking the potential of stem cell therapies (Hashimoto *et al.* 2018). However, the challenges in predicting the precise type and timing of these transitions persist due to the intricate and dynamic nature of biological systems, alongside the added complexity introduced by the high-dimensional nature of the data.

In response to these challenges, we introduce FateNet, a novel framework that integrates dynamical systems theory with deep learning to discern when cells make decisions and predict the type of transition the system is approaching. For a deep learning classifier to be effective and applicable across diverse scenarios, it necessitates training on a broad spectrum of data. We generate time series training data using simulations from a comprehensive library of different dynamical system models that possess various types of bifurcation. The universal properties of bifurcations, manifested in time series as a system approaches a bifurcation, facilitate this generalizability. To validate our framework, we conduct extensive testing using both simulated and biological data, spanning different dataset sizes and varying noise levels. For the hematopoietic data, predictions were made on down-sampled trajectories, showing that FateNet can work with missing data. Since FateNet predicts the type of bifurcation, it can identify genes that can prevent harmful bifurcations in a system and promote favorable transitions. Here, by performing both hard (knock-out) and soft (over-expression) interventions to a developmental system we have shown the possibility of targeting a specific set of genes to promote, prevent, or modify the type of transition in hematopoiesis.

FateNet is a first attempt to combine dynamical systems theory with deep learning to predict the type of bifurcation resulting in a cell fate transition. It is trained to predict fold, pitchfork and transcritical bifurcations, which have been implicated in various cell fate transitions (Huang *et al.* 2007, Ferrell 2012, Matsuda *et al.* 2015, Bargaje *et al.* 2017). However, there exist other types of bifurcations that may also be involved with cell fate transitions, such as the Hopf bifurcation (Hat *et al.* 2016) and global bifurcations (Raju and Siggia 2024). Future developments will involve a training library that includes a larger number of bifurcation types. FateNet is also currently restricted to univariate data, which is obtained from the first principal component. A boost in performance could be obtained by training the classifiers on multivariate data, allowing them to interpret multiple components of the PCA, where potentially more information about the bifurcation could be obtained. Recent success in predicting critical transitions using network measures (Zhong *et al.* 2021, 2022, 2023) suggests that training classifiers in a similar way on gene interaction networks could be fruitful. Finally, it could be beneficial to determine the time series features that FateNet uses to determine the type of bifurcation using interpretability methods such as layer-wise relevance propagation (Bach *et al.* 2015). It seems plausible that it is learning features such as critical slowing down and distinguishable changes in the stability landscape to determine the type of bifurcation.

We believe future studies can benefit from the application of FateNet to compare bifurcation types in biological systems, particularly when comparing differences in the type and timing of system transitions in healthy and diseased conditions. Many disorders, including cancer and fibrosis, can be perceived as distinct types of transitions within a biological system (Korolev *et al.* 2014, Kembro *et al.* 2018, Glass and Mackey 2020, Sadria *et al.* 2022b). Consequently, FateNet provides valuable insights into identifying effective targets for interventions and optimal timing in guiding and redirecting the system’s evolution from an impaired to a healthy state.

## Supplementary Material

btae525_Supplementary_Data

## Data Availability

The simulated scRNA-seq data can be generated by SERGIO and can be found at: https://github.com/PayamDiba/SERGIO. The hematopoiesis Weinreb *et al.* data can be downloaded from Gene Expression Omnibus (GEO) under accession number GSE140802. The dataset of pancreatic development can be found from GEO under accession number GSE132188. The simulated scRNA-seq data generated by the simple gene regulatory network can be found in the GitHub repository https://github.com/ThomasMBury/fatenet.
